# Land Cover and Rainfall Interact to Shape Waterbird Community Composition

**DOI:** 10.1371/journal.pone.0035969

**Published:** 2012-04-27

**Authors:** Colin E. Studds, William V. DeLuca, Matthew E. Baker, Ryan S. King, Peter P. Marra

**Affiliations:** 1 Smithsonian Conservation Biology Institute, Migratory Bird Center, National Zoological Park, Washington, D. C., United States of America; 2 Department of Environmental Conservation, University of Massachusetts, Amherst, Massachusetts, United States of America; 3 Department of Geography and Environmental Systems, University of Maryland, Baltimore, Maryland, United States of America; 4 Center for Reservoir and Aquatic Systems, Department of Biology, Baylor University, Waco, Texas, United States of America; National Institute of Water & Atmospheric Research, New Zealand

## Abstract

Human land cover can degrade estuaries directly through habitat loss and fragmentation or indirectly through nutrient inputs that reduce water quality. Strong precipitation events are occurring more frequently, causing greater hydrological connectivity between watersheds and estuaries. Nutrient enrichment and dissolved oxygen depletion that occur following these events are known to limit populations of benthic macroinvertebrates and commercially harvested species, but the consequences for top consumers such as birds remain largely unknown. We used non-metric multidimensional scaling (MDS) and structural equation modeling (SEM) to understand how land cover and annual variation in rainfall interact to shape waterbird community composition in Chesapeake Bay, USA. The MDS ordination indicated that urban subestuaries shifted from a mixed generalist-specialist community in 2002, a year of severe drought, to generalist-dominated community in 2003, of year of high rainfall. The SEM revealed that this change was concurrent with a sixfold increase in nitrate-N concentration in subestuaries. In the drought year of 2002, waterbird community composition depended only on the direct effect of urban development in watersheds. In the wet year of 2003, community composition depended both on this direct effect and on indirect effects associated with high nitrate-N inputs to northern parts of the Bay, particularly in urban subestuaries. Our findings suggest that increased runoff during periods of high rainfall can depress water quality enough to alter the composition of estuarine waterbird communities, and that this effect is compounded in subestuaries dominated by urban development. Estuarine restoration programs often chart progress by monitoring stressors and indicators, but rarely assess multivariate relationships among them. Estuarine management planning could be improved by tracking the structure of relationships among land cover, water quality, and waterbirds. Unraveling these complex relationships may help managers identify and mitigate ecological thresholds that occur with increasing human land cover.

## Introduction

Conversion of natural habitats to human dominated landscapes has led to worldwide deterioration of estuarine ecosystems [1]. In many regions, the continued spread of human land cover has been accompanied by greater variation in climatic conditions, such as strong cycles of drought and precipitation [2]. Together, human land cover and rainfall shape estuarine condition directly by reducing coastal habitat quality or indirectly by lowering water quality. Both agriculture and urban development directly impair estuaries through degradation or loss of coastal wetlands, modification of other natural shoreline areas, and habitat fragmentation and isolation [3]–[5]. These land cover types also indirectly degrade estuaries by carrying nutrients and contaminants from terrestrial watersheds into coastal water bodies. Rainfall events amplify the hydrological connectivity between watersheds and estuaries, potentially leading to severe eutrophication [6], [7]. With watersheds in many coastal areas undergoing dynamic changes in land cover and climate, management and restoration programs could benefit from understanding how rainfall interacts with expanding human development to shape estuarine condition.

The greatest risk posed by eutrophication of coastal waters is the decrease in dissolved oxygen (DO) when blooms of aquatic algae die and are consumed through microbial respiration [8]. Hypoxia occurs when DO falls below 2 mg/L and has been shown to cause mortality of sessile benthic invertebrates and trigger emigration of commercially harvested crab and fish species to more oxygen-rich waters [9], [10]. Despite these well-documented responses of lower trophic level organisms, the consequences for higher order consumers are essentially unknown. Because conservation of upper trophic level wildlife is mandated by state and federal management agencies, there is a need to understand how these species respond to eutrophication and other disturbances associated with increasing human land cover.

Bird communities can be robust indicators of biological condition because they often occupy the highest trophic level in an ecosystem, causing them to integrate the effects of abiotic stressors acting on species at lower trophic levels [11], [12]. DeLuca et al. [13] developed an index of waterbird community integrity in Chesapeake Bay, USA and evaluated its sensitivity to anthropogenic disturbance. They found that even low levels of urban development, particularly when located close to the estuarine shoreline, severely impaired the waterbird community. This finding strongly suggests that waterbird communities are directly degraded by urban development, but, because this study did not assess potential indirect pathways that compromise water quality, the underlying causes remain unclear. Waterbird abundance depends on both habitat quality and food availability [14]. Therefore, waterbird communities should also be sensitive to changes in water quality because these species primarily consume fish and invertebrates, some of whose local abundances decline when eutrophic conditions prevail [15].

In 2002 and 2003, we monitored the waterbird community, nitrate-N concentrations, dissolved oxygen levels, and salinity in 27 subestuaries of Chesapeake Bay. In 2002, a severe region-wide drought limited freshwater flow and nutrient inputs to the Bay to near historic lows [16], [17]. In contrast, the Bay received more than twice the amount of freshwater in 2003 and the third highest amount since 1937, much of which was concentrated in the spring and summer months. This massive influx nearly tripled nitrogen export from the previous year, resulting in sustained hypoxia throughout the estuary [16]. Here, we explore how this temporal variation in rainfall interacted with spatial variation in land cover to shape waterbird community composition. We used non-metric multidimensional scaling and structural equation modeling to evaluate direct and indirect effects of land cover on waterbird community composition and to assess change in their strength between years. We predicted waterbird community composition would be limited directly by urban development in both years, and indirectly by lower water quality due to greater hydrological connectivity between watersheds and subestuaries in 2003.

## Methods

**Figure 1:**
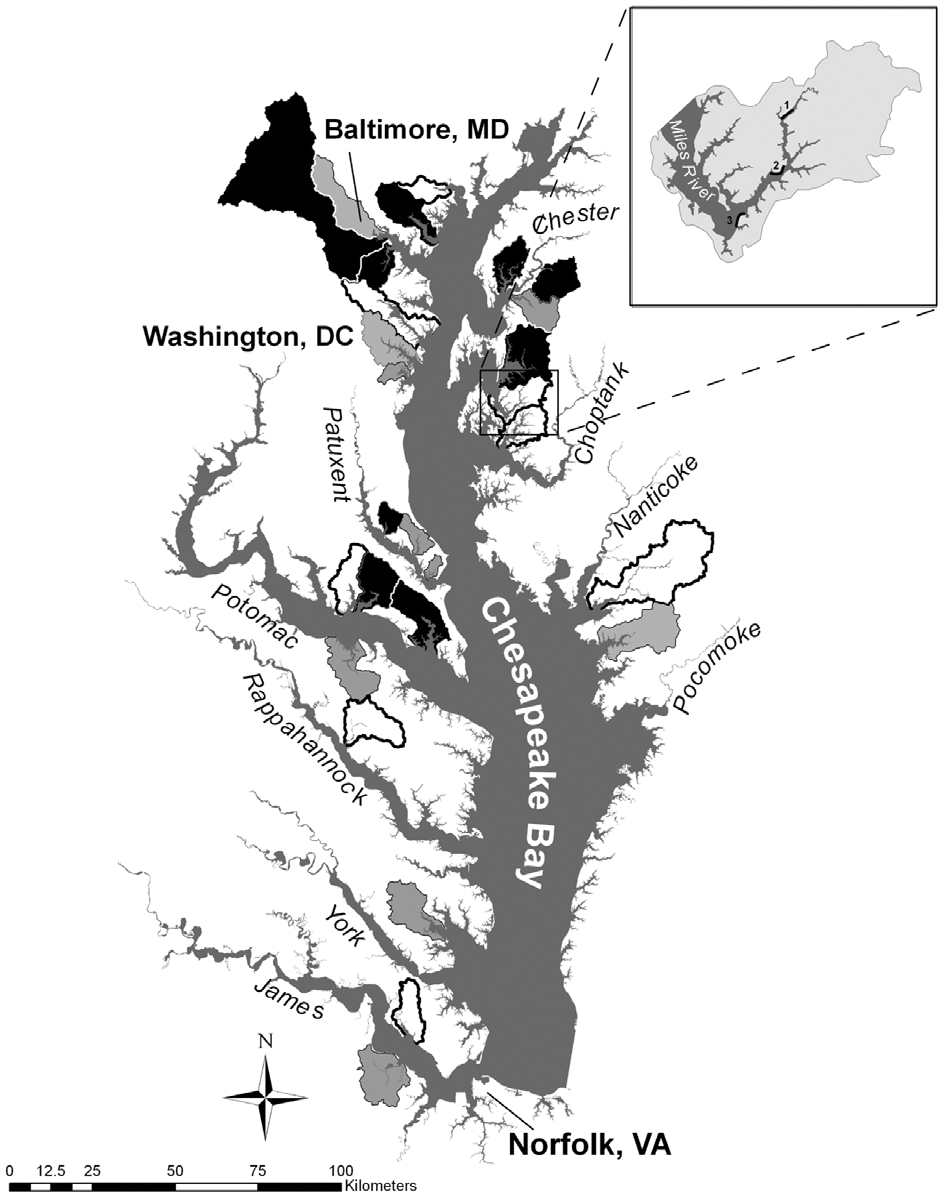
Distribution of 27 watersheds and associated subestuaries in Chesapeake Bay, USA where relationships between land cover, water quality, and waterbird communities were studied in the dry year of 2002 (white), the wet year of 2003 (gray), and in both years (black). The inset shows an example distribution of the three waterbird sampling transects in the lower, middle, and upper reaches of the subestuary.

Fieldwork was conducted from 2002–2003 in 27 subestuaries of Chesapeake Bay, USA (39° 23′ N–36° 48′ N, 76° 45′ W–75° 44′ W). Each subestuary had a distinct embayment that separated it from major tributaries of the Bay and a watershed drained by a third to fifth order stream [18]. We quantified land cover within each watershed by using ArcGIS software (ESRI, Redlands, CA, USA) and the 1992 National Land Cover Database derived from 30-m resolution Landsat Thematic mapper images [19]. We calculated the percentage watershed area covered by agriculture and urban development and the percentage area of emergent marsh within 500 m of the shoreline. We summarized the percentage of urban development in each watershed weighted by its squared inverse distance (IDW) to the shoreline because of past evidence that ecological indicators in Chesapeake Bay are sensitive to this land cover in close proximity to subestuaries [13], [18]. We sampled 17 subestuaries in 2002, 20 in 2003, and a subset of nine in both years (Fig. 1). This design permitted us to balance spatial coverage and temporal replication of data collection.

We surveyed the waterbird community along three, 1-km transects in each subestuary. Transects were positioned in the upper, middle, and lower thirds of subestuaries and were oriented parallel to and 100 m from the shoreline (Fig. 1 inset). Adjacent transects within subestuaries were separated by >500 m to reduce the probability of counting an individual more than once. We surveyed waterbirds with the double observer approach from a boat traveling at three knots along transects [20]. For this study, we defined waterbirds as all non-passerine bird species that forage exclusively or opportunistically on aquatic estuarine organisms (e.g., gulls, terns, waders, raptors, kingfishers, and waterfowl). Observers counted all individuals on the water, in the air, or perched along the shoreline within 100 m of transects. We surveyed each transect three times from 15 May–15 August between 0600 h and 1300 h and used program DOBSERV to calculate abundance estimates corrected for imperfect detection probabilities [20]. Deluca et al. [13] incorporated these corrected abundance estimates along with information about foraging and nesting niche breadth, migratory behavior, and regional rarity into an index of waterbird community integrity for each subestuary. We employed this index in the present study to characterize spatial and temporal variation in waterbird community composition (WCC) because subestuaries with high scores supported high abundances of specialist species with high conservation value and those with lower scores harbored high abundances of generalist species with lower conservation value (see Text S1 for index development).

Six water quality sampling stations were distributed via a random sampling approach nearby to waterbird survey transects in each subestuary. Salinity, dissolved oxygen (DO), and nitrate-N were measured approximately 50 m from shore at two locations at every sampling station. Salinity (ppt) and DO (mg/L and percent saturation) were measured using an YSI 556 multiparameter instrument (YSI Inc., Yellow Springs, OH, USA) at 10 cm below the water surface and 10 cm above the bottom. The difference between percent saturation of DO at the surface and bottom (DO difference) was used as a metric of potential benthic hypoxia. This index also helped alleviate differences in DO due to diel fluctuations among sampling stations because measurements were not collected at the same time of day across all stations. Nitrate-N (µg/L) samples were collected near the water surface in acid-washed polyethylene bottles, stored on ice, and returned to the laboratory for analysis [21]. No specific permits were required for the described field studies.

We used non-metric multidimensional scaling (MDS) to describe spatial and temporal variation in the abundance of generalist and specialist species in each subestuary following steps outlined by [22]. We used corrected abundance estimates of each species to compute Bray-Curtis distances among subestuaries for each year separately. The number of MDS axes was chosen by minimizing stress, a measure of the mismatch between distance among species indicated by the Bray-Curtis matrix and distance in the ordination. Species centroids were mapped in ordination space by weighted averaging. We used rotational vector fitting to relate land cover and water quality indices to the ordination [23]. Significance of vectors was estimated using 1000 random permutations. Ordinations and vector fitting were performed in program R 2.11 using the vegan package [24], [25].

We used structural equation modeling (SEM) to investigate a set of hypothesized causal relationships among land cover, water quality, and WCC. These relationships can be visualized through a path model in which arrows indicate the proposed effect of one variable on another. The path diagram was drawn based on past research from Chesapeake Bay and on established linkages between land cover and estuarine condition [13], [21], [26]. Direct paths were expected to act on WCC as a function of the amount of emergent marsh habitat and urban development in each subestuary [13]. Subestuaries with a high percentage of emergent marsh were predicted to have high WCC scores because many waterbirds forage in this habitat, and it serves as a nursery for many fish species consumed as prey. Subestuaries adjacent to watersheds with a high percentage of urban land cover were hypothesized to have low WCC scores due to degradation of marsh and shoreline habitat. Such an effect could occur through habitat fragmentation and isolation, habitat alteration from shoreline hardening, or prevalence of invasive vegetation [3], [18], [27].

Indirect pathways proceeding from urban development and agriculture were hypothesized to act on WCC through their effect on water quality. Agriculture and urban development are important determinants of nitrate-N and DO levels in estuaries [28]. Subestuaries with a high percentage of these land cover types in nearby watersheds were predicted to have low WCC scores owing to high nitrate-N concentrations and large DO differences. The model posited that these two indices of water quality influence a conceptual and unmeasured latent variable labeled as “Eutrophication”. We included a measure of surface salinity in the model to index the spatial position of subestuaries on the landscape. Freshwater inputs are higher in northern compared to southern reaches of Chesapeake Bay, and such gradients can lead to spatial variation in nutrient concentration in estuaries [29], [30]. At the northern end of the Bay, the Susquehanna River is a major source of freshwater, and agricultural discharges to this river account for a substantial fraction of nitrogen loads to the Bay [31]. Relationships among land cover and salinity were drawn using double-headed arrows because these variables often covary with one another, particularly in this landscape [32].

We used multi-group sampling to identify the path model that best fit annual variation in the data that could have occurred due to differences in rainfall. Multi-group sampling allows parameters of interest to be constrained to equality for subsets of data, thus permitting evaluation of a priori hypotheses [33]. In the first model, we allowed all paths to vary between years to test the hypothesis that annual variation in the data occurred due to differences in both direct and indirect effects. The second model tested the hypothesis that annual variation in the data was driven by differences in degradation of marsh and coastal habitat by requiring only indirect paths to be equal. In the third model, we fixed only the direct paths to be equal to test the hypothesis that data varied between years due to changes in water quality. The final model imposed equality constraints on all paths to test the hypothesis that there was no discernable annual variation in the data.

The path coefficients in each model are values that maximize the likelihood of the covariance structure in the data given the covariance structure proposed by the path model. We assessed the fit of each model with a chi-squared goodness-of-fit test, where a significant chi-squared indicates that the model does not fit the data. This statistic is an asymptotic approximation of a chi-squared distribution when the data follow a multivariate normal distribution. We also assessed model fit with the comparative fit index (CFI) and root mean square error (RMSEA), two commonly used indices that provide an approximate measure of model fit. Models with a CFI >0.95 and a RMSEA of <0.05 indicate a good fit to the data [34]. Because our sample size was smaller than desired for structural equation models estimated through maximum likelihood, we performed Monte Carlo permutation tests with 1000 replicates to evaluate the robustness of each model fit. Significance of path coefficients for the best model was judged based on an empirical distribution generated from 1000 bootstrap replicates of the data. All SEM analyses were done with AMOS 19.0 [35].

We used least squares regression to evaluate temporal change in the relationship among land cover, water quality, and WCC for the subset of subestuaries studied in both years. For this analysis, we considered only predictors judged important in the best-fit model from the SEM multi-group analysis. We expected statistical inference from this analysis might be somewhat limited given that only nine subestuaries were studied in both years. However, our primary goal in this analysis was to assess concordance between regression coefficients from this model and path coefficients from the best-fit multi-group model that were fit with data from all subestuaries. Regression analyses were done with program R 2.11 [24].

## Results

**Figure 2:**
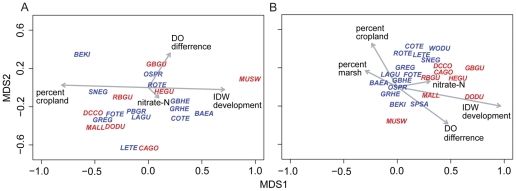
Non-metric multidimensional scaling (MDS) of Chesapeake Bay, USA waterbird communities in (A) 17 subestuaries in the drought year of 2002 (stress  = 0.176) and (B) 20 subestuaries in the near record wet year of 2003 (stress  = 0.183). Species centroids were mapped in 2-dimensional ordination space and rotational vector fitting was used to relate land cover, water quality, and waterbird community composition (WCC) scores to the ordination. WCC scores are a measure of waterbird community composition, where subestuaries with high abundances of specialist species (blue) received high scores and those with high abundances of generalists (red) received lower scores (DeLuca et al. 2008). Species codes and WCC scores are shown in Table S1. IDW development is the percent urban development in each watershed weighted by the square of its inverse distance to the shoreline. The vector for percentage marsh in 2002 was short and was not plotted for clarity.

The MDS ordination of WCC revealed distinct inter-annual patterns in the distribution of generalist and specialist species across the 27 subestuaries. Two-dimensional solutions were chosen because stress was relatively low for both years (2002: stress  = 0.176; 2003: stress  = 0.183). Both generalists and specialists were widely distributed among subestuaries in the drought year of 2002 and demonstrated no clear association with land cover or water quality indices (Fig. 2A, see Table S1 for species names and WCC scores). Conversely, when nutrient flow to the Bay reached a near record high in 2003, generalist species exhibited a pronounced shift toward subestuaries in developed watersheds with high nitrate-N concentrations and large differences between surface and bottom DO, whereas specialists exhibited a weaker but opposite trend (Fig. 2B).

The multi-group SEM indicated strong annual variation in the paths affecting WCC. The model in which all paths were unconstrained and thus required to vary between years provided the best fit to the data (χ^2^ = 11.49, *df* = 12, *P* = 0.487; RMSEA<10^−3^; CFI = 1.00). The model that fixed direct paths to be equal between years provided a reasonable fit to the data based on the goodness of fit test, but CFI and RMSEA values indicated somewhat poor fit (χ^2^ = 17.40, *df* = 14, *P* = 0.236; RMSEA  = 0.09; CFI  = 0.96). Models that required either indirect paths or all paths in the model to be equal between years fit the data poorly (χ^2^ = 27.72, *df* = 19, *P* = 0.089; RMSEA  = 0.12; CFI  = 0.90 and χ^2^ = 35.33, *df* = 21, *P* = 0.026; RMSEA  = 0.14; CFI  = 0.83, respectively). Monte Carlo permutation tests indicated that these estimates of model fit were robust (all models P<0.05). Examination of multivariate kurtosis values revealed moderate departure from multivariate normality (2002: kurtosis  = 6.48, critical ratio  = 1.12; 2003: kurtosis  = 8.11, critical ratio  = 1.62), but a Bollen-Stine bootstrap indicated that model fit was not compromised (*P* = 0.878). Based on these results, we interpreted the model in which all paths varied independently between 2002 and 2003. Pearson correlation coefficients, variances, and covariances among variables in these models are presented in Table S2.

Observed correlations among predictors showed strong concordance with those implied by the model (Table 1). These relationships suggested that differences between observed correlations and total effects resulted from non-causal or spurious correlations among predictors, most likely due to unanalyzed relationships or residual spatial autocorrelation that could not be accounted for by covariance relationships specified in the path model. Still, several predictors exhibited differences between their total effects and observed correlations with WCC. In particular, surface salinity (2002) and percent cropland (2002 and 2003) had moderate positive correlations with WCC, but had total effects that changed sign or were notably lower (Table 1). Conditioning of predictors by SEM, however, indicated that these correlations were enhanced partially by strong negative associations with urban development (Table 1). Discrepancies in how land cover acted on DO difference were also apparent. Observed correlations, implied correlations, and total effects between percentage cropland and DO difference were consistently negative, whereas those between percentage development and DO difference were uniformly positive, an outcome most likely attributable to the strong negative correlation between these land cover types and differences in their pollutant export dynamics (Table 1, Table S2).

**Table 1:** Standardized total effects, model correlations, and observed correlations from the best-fit structural equation model (SEM) in which all paths were free to vary between the drought year of 2002 and the wet year of 2003.

			Response		
Year	Predictor	Analysis	Nitrate-N	DO difference	WCC
**2002**	**Salinity**	Total effects	**−0.437**	–	−0.086
		Model *r*	**−0.617**	−.258	0.230
		Observed *r*	**−0.617**	−.320	**0.360**
	**Cropland**	Total effects	0.092	−0.061	0.022
		Model *r*	−0.127	**−0.418**	**0.435**
		Observed *r*	−0.127	**−0.418**	**0.393**
	**Development**	Total effects	0.447	**0.665**	**−0.735**
		Model *r*	0.570	**0.698**	**−0.721**
		Observed *r*	0.570	**0.698**	**−0.715**
	**Marsh**	Total effects	–	–	0.332
		Model *r*	−0.127	−0.119	0.438
		Observed *r*	−0.041	−0.246	0.458
		Multiple *r* ^2^ for prediction	**0.513**	**0.490**	**0.652**
**2003**	**Salinity**	Total effects	**−0.538**	–	**0.149**
		Model *r*	**−0.709**	−0.012	**0.466**
		Observed *r*	**−0.709**	−0.197	**0.563**
	**Cropland**	Total effects	0.220	**−0.371**	−0.009
		Model *r*	0.188	**−0.533**	0.290
		Observed *r*	0.188	**−0.533**	**0.393**
	**Development**	Total effects	**0.364**	0.345	**−0.667**
		Model *r*	**0.429**	**0.519**	**−0.769**
		Observed *r*	**0.429**	**0.519**	**−0.768**
	**Marsh**	Total effects	–	–	0.257
		Model *r*	−0.285	−0.116	0.438
		Observed *r*	−0.105	**−0.319**	0.458
		Multiple *r* ^2^ for prediction	**0.579**	**0.377**	**0.652**

Differences between observed and model correlations give specific estimates of residual error in model fit. Total effects are the sum of all direct and indirect effects between predictor and response variables, where indirect effects are the product of all direct effects along a hypothesized casual pathway. Coefficients in bold differed from 0 based on 1000 bootstrap replicates.

**Figure 3:**
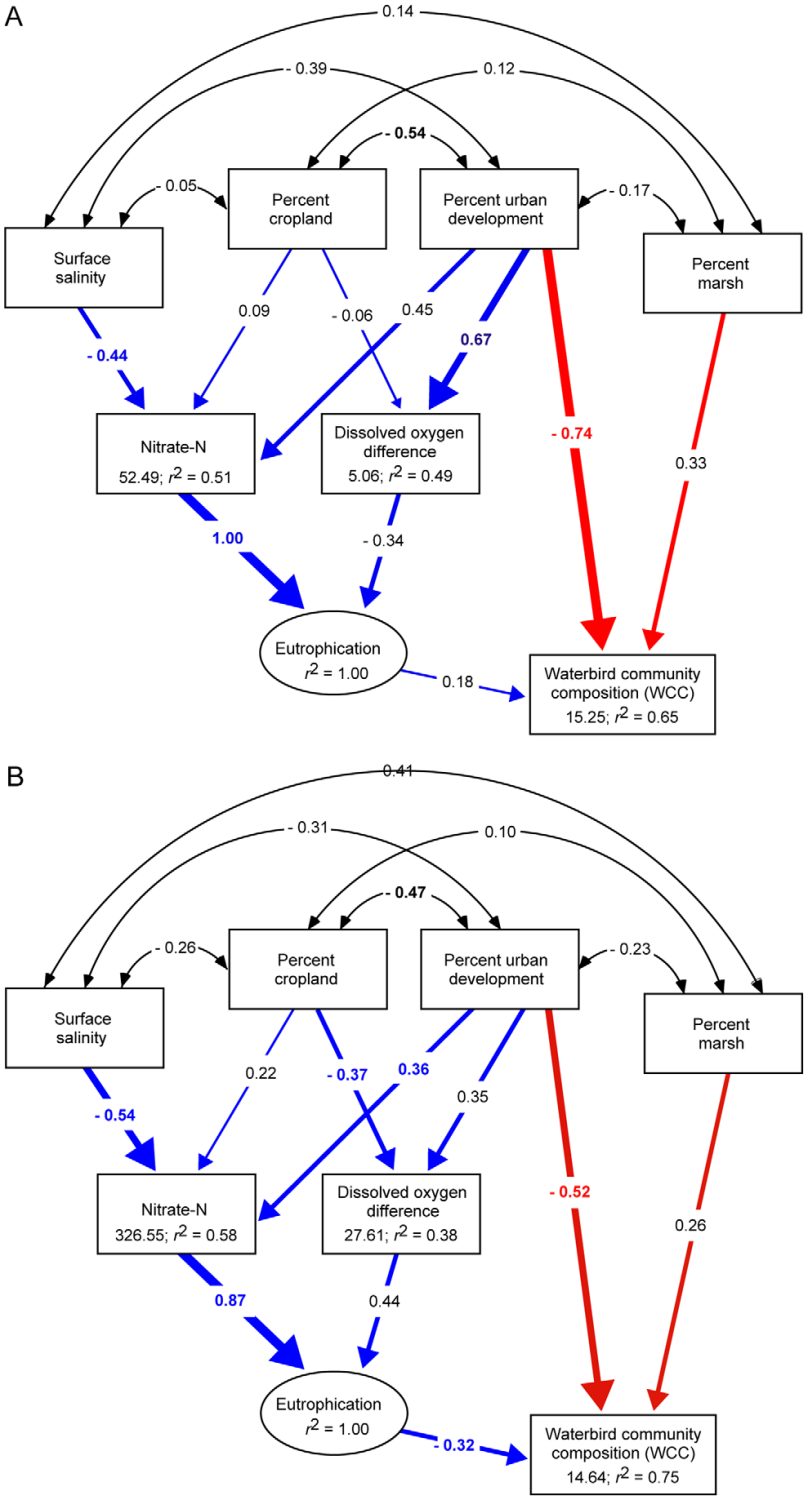
Structural equation models (SEM) testing hypothesized causal effects of land cover and rainfall on waterbird community composition (WCC) in Chesapeake Bay, USA. The best-fit model indicated variation in the strength of direct (red) and indirect paths (blue) between (A) 2002, a year of severe drought, and (B) 2003, a year of near record rainfall (χ^2^ = 11.49, *df* = 12, *P* = 0.487; RMSEA <10^−3^; CFI  = 1.00). Values between variables are standardized path coefficients. Arrow widths are proportional to the size of path coefficients, but only values in color differed from zero based on a parametric bootstrap of the data with 1000 replicates. Estimated intercepts and the proportion of variance explained by that part of the model appear below variable names. IDW development is the percent urban development in each watershed weighted by the square of its inverse distance to the shoreline.

Fitted path coefficients indicated substantial annual variation in how land cover and water quality shaped WCC, but also revealed a consistent negative effect of urban development. In the dry year of 2002, only the direct effect of urban development was a strong predictor of WCC (Fig. 3A). WCC scores were lower in subestuaries with a high percentage of urban development (λ = −0.74, *P* = 0.046). The difference in percent saturation of DO between the surface and bottom of the water column was greater in subestuaries with more urban development (β = 0.67, *P* = 0.009), but did not markedly increase the estimated eutrophication variable (β = −0.34, *P* = 0.596). Nitrate-N levels were higher in subestuaries with lower salinity, reflecting elevated concentrations in relatively fresh northern compared to brackish southern subestuaries (λ = −0.44, *P* = 0.048). This trend led to greater predicted values of the latent eutrophication variable (β = 1.00, *P* = 0.002), but it was not substantial enough to alter WCC (β = 0.18, *P* = 0.666).

In the wet year of 2003, WCC again depended on the direct effect of urban development but also on the indirect effects of urban development and salinity on water quality. WCC scores remained lower in subestuaries with a high percentage of urban development (λ = −0.52, *P* = 0.047; Fig. 3B). Nitrate-N concentrations were greater in subestuaries surrounded by a high percentage of urban development (λ = 0.36, *P* = 0.043), and in those with lower salinity (λ = −0.54, *P* = 0.021), indicating elevated nutrient levels in northern compared to southern subestuaries. Together, these effects were associated with a mean nitrate-N concentration in subestuaries over six times greater than in the previous year and a marked rise in predicted values of the latent eutrophication variable (β = 0.87, *P* = 0.002; Table 2). DO difference was greater in subestuaries adjacent to watersheds with a lower percentage of cropland (λ = −0.37, *P* = 0.049), however this negative pattern also reflected a large DO difference in subestuaries with a large amounts of urban development (Table 1). This latter relationship is more consistent with predicted responses of DO to land cover and with the observed deterioration in mean water quality from 2002 to 2003 (Table 2, Table 3). In contrast to 2002, predicted eutrophication in 2003 was strong enough to alter WCC (β = −0.32, *P* = 0.047), a change implied by the MDS ordination to include a decrease in the abundance of specialist species and an increase in generalists.

**Table 2:** Water quality indices (mean ± SE) from six sampling stations in each of 27 subestuaries of Chesapeake Bay, USA.

	Year	
Water quality	2002	2003
Salinity (ppt)	10.58±1.17	5.48±0.59
Nitrate-N (μg/L)	27.41±13.46	169.53±54.72
Bottom DO (% saturation)	65.69±4.51	52.01±4.61
Minimum bottom DO (% saturation)	41.07±4.04	28.46±4.54
Hypoxia frequency (prop. of stations)*	0.08	0.18

* Hypoxia frequency is the proportion of stations across all subestuaries where bottom DO concentration was <2 mg/L.

**Figure 4:**
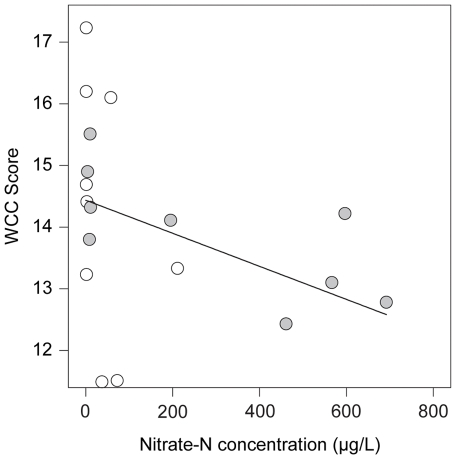
Relationship between nitrate-N concentration and waterbird community composition (WCC) in the nine subestuaries of Chesapeake Bay, USA that were sampled in both the drought year of 2002 and the wet year of 2003. Nitrate-N levels did not limit waterbird community composition during the drought year of 2002 (white; *r* ^2^ = 0.12. *P* = 0.368), but were associated with degraded waterbird communities during the wet year of 2003 (grey; *r* ^2^ = 0.51. *P* = 0.032).

Annual variation in WCC associated with elevated nitrate-N concentration in subestuaries was also evident when we considered this relationship in only the subset of nine subestuaries that were studied in both years. During the dry year of 2002, WCC did not vary in relation to nitrate-N levels (Fig. 4; *r*2 = 0.12. *P* = 0.368). In contrast, in the wet year of 2003, WCC scores in the same subestuaries decreased with increasing nitrate-N concentration (*r*2 = 0.51. *P* = 0.032). These data suggest that higher nitrate-N concentrations observed in 2003 were unlikely due to spatial variation resulting from sampling a group of different subestuaries in 2002 and 2003.

**Table 3:** Dissolved oxygen indices (mean ± SE) from six sampling stations in each of a subset of 19 subestuaries where percent cropland or IDW development covered >15 percent of the surrounding watershed in Chesapeake Bay, USA.

		Land cover	
Year	Dissolved oxygen	Cropland	IDW development*
**2002**	Surface DO (% saturation)	73.61±3.64	91.26±12.15
	Bottom DO (% saturation)	66.00±3.53	63.48±11.87
	DO difference (% saturation)	7.61±2.62	27.78±10.51
**2002**	Surface DO (% saturation)	73.66±5.28	92.24±10.53
	Bottom DO (% saturation)	54.70±7.11	49.56±8.29
	DO difference (% saturation)	18.96±4.84	42.67±7.55

* IDW development is the percent urban development in the watershed weighted by its squared inverse distance from the shoreline.

## Discussion

Our results support the hypothesis that increased hydrological connectivity between the terrestrial landscape and the aquatic environment can lower water quality enough to alter the composition of estuarine waterbird communities. The MDS ordination indicated that urban watersheds shifted from a mixed generalist-specialist community in 2002 to generalist-dominated community in 2003. The multi-group SEM revealed that this change in WCC was associated with higher overall trophic status of subestuaries as indicated by elevated nitrate-N concentrations. High nitrate-N concentrations in estuarine grab samples can indicate that nitrogen demand has been temporarily satisfied, that primary producers have yet to respond to continued enrichment, or both [28]. Even though our models did not incorporate rainfall or nitrogen loads explicitly, concurrent research, including long-term monitoring of Chesapeake Bay water quality, provides independent evidence that higher rainfall in 2003 facilitated a strong increase in nitrogen loading compared to 2002 [16], [17], [36]. Together, these data imply that continued expansion of urban development and strong rainfall events that flush accumulated pollutants from the landscape may interact to promote estuarine waterbird communities increasingly dominated by generalist species.

High predicted eutrophication in the wet year of 2003 likely represents elevated nitrate-N discharge from at least three distinct terrestrial sources that were differentiated from one another only after accounting for spatial autocorrelation among land cover types. Agricultural lands, and cropland in particular, typically supply the majority of nutrients to estuaries [21], [37], [38]. The Susquehanna River, situated at the northern end of the Bay, drains a region of extensive agriculture and accounts for a large fraction of annual nitrogen loads [31]. The increase in nitrate-N concentrations in lower salinity northern subestuaries probably reflects lagged nutrient discharges from the Susquehanna River that originated from distant, upstream cropland, potentially as far north as New York. Conversely, local cropland in watersheds surrounding subestuaries appeared to have little effect on measured nitrate-N concentrations. One explanation for this pattern is that nitrogen export from cropland peaks in early spring before green vegetation emerges and evapotranspiration reduces flow rates of smaller rivers [16]. Nitrate-N arriving to subestuaries in spring would have been converted to algal biomass and organic detritus by the time water our quality sampling occurred. Nitrate-N enrichment from local cropland therefore was detectible primarily through its effect on bottom DO, whereas DO difference remained largely unresponsive to increasing cropland (Table 3). Unlike those dominated by agriculture, urban watersheds have comparatively lower evapotranspiration and appeared to load similar amounts of nitrate-N throughout the summer, leading to greater observed concentrations, supersaturation in surface DO, pronounced DO difference, and high predicted values of the latent eutrophication variable (Table 3). Therefore, the negative correlation between DO difference and cropland likely was driven by DO trends in urban watersheds and the negative correlation between cropland and development that exists in this region [32].

We did not measure the response of lower trophic level organisms to eutrophication, but it is likely that the increase in generalist waterbird species in 2003 reflected disturbance propagating from the base to the top of the estuarine food web [39], [40]. All of the specialists recorded in this study prey on fish species that concentrate near estuarine wetlands during the summer breeding season, whereas half of the generalists observed (mute swan [*Cygnus olor*], mallard [*Anas platyrhynchos*], domestic duck [*Anas* and *Cairina spp.*], and Canada goose [(*Branta canadensis*]) incorporate aquatic or terrestrial plants in their diet. Eutrophic, oxygen depleted conditions, such as those present in 2003, have been linked to reduced abundance and mortality of fish species consumed as prey by piscivorous waterbirds in Chesapeake Bay and other shallow estuaries [41]–[43]. Because some waterbirds travel long distances between breeding and foraging areas, their distribution while foraging should reflect the availability of prey and the overall quality of estuarine habitat [44], [45]. Thus, it is possible that the sensitivity of WCC to the annual change in water quality was due to piscivorous species tracking food resources to areas minimally affected by eutrophication and hypoxia. Low salinity in estuaries may also reduce fish abundance through temporary emigration to more saline waters [46]. Lower salinity in 2003 could therefore have facilitated the increase in generalist waterbird species, particularly in urban subestuaries at northern reaches of the Bay (Table 2). Nonetheless, the total effect of salinity on WCC was less than half that of nitrate-N in 2003, suggesting that nutrient loading was the primary aspect of water quality affecting waterbirds (Table 1; Fig. 3B).

Two lines of evidence suggest that we were successful at capturing variation in water quality across years and that the observed effects were not due to spatial variation caused by sampling different subestuaries in each year. First, the intercept estimated for nitrate-N concentration in the best-fit model was more than six times greater in 2003 compared to 2002 (Table 2). This difference is in line with annual variation in nitrate-N concentration in small streams and major rivers entering Chesapeake Bay found by other studies during the same time period [16], [36]. Second, annual variation in the relationship between nitrate-N and WCC in the subset of nine subestuaries studied in both 2002 and 2003 closely paralleled the trend found in the full data set, which included sites surveyed in only one of two years.

Even though elevated nitrate-N concentrations in 2003 were linked to a strong change in WCC, urban development directly limited the waterbird community in both years, an outcome likely due in part to fragmentation and loss of both near-shore terrestrial and wetland habitat. Fragmentation of terrestrial shoreline habitat by human development can reduce habitat suitability for piscivorous species that use shoreline foraging or nesting perches (e.g., belted kingfisher [*Ceryle alcyon*] and osprey [*Pandion haliaetus*]) or prefer unbroken stretches of natural shoreline (e.g., great egret [*Ardea alba*] and green heron [*Butorides virescens*]). In addition, shoreline modification can facilitate colonization of wetlands by terrestrial and invasive vegetation, especially *Phragmite*s *australis* [18]. This species can fragment native vegetation and render even large wetlands inhospitable to foraging waterbirds. Moreover, fragmentation and isolation of natural vegetation by urban development may facilitate increases in mammalian predators and limit nesting habitat suitability, potentially forcing waterbirds to commute longer distances to foraging areas.

Some unknown portion of the variation we have attributed to direct pathways could also have occurred through unmeasured aspects of indirect pathways, including phosphorus export, sewage overflow, and the presence of toxins [47]–[49]. Nitrate-N concentrations in subestuaries increased with the percentage developed land in watersheds not only in the wet year 2003, but also in the absence of substantial rainfall in 2002 (Fig. S1). This pattern is consistent with a greater contribution of distinct point sources of nitrate-N with high amounts of urban development in the dry year of 2002. Because urban areas can have point sources that do not require strong rainfall events to deliver nutrients to estuarine environments (e.g., water treatment facilities, sewage leaks), it is probable that they were responsible some of the negative effect of urban development on WCC, especially in the drought year of 2002. Urban environments also contain sources of nitrogen that may be continually replenished throughout the growing season (e.g., lawn fertilizers, atmospheric deposition). Increasing development has been shown to shift the mode of nitrogen export from base flows toward event flows, so flushing of these sources likely increases with more intense precipitation events [36], [50].

The expansion of human development in the Chesapeake Bay watershed is likely to result in greater nutrient inputs and enhanced conditions for foraging generalists, a situation that could be exacerbated by predicted increases in severe storms [2]. These changes may not only reduce foraging habitat quality for piscivorous specialists, but could also limit their populations through increased competition and predation by generalists. Agonistic interactions with double-crested cormorants (*Phalacrocorax auritus*) can reduce reproductive success of other colonial nesting species, and have been implicated in displacement of heron and egret rookeries [51], [52]. Cormorants can also severely deplete fish populations, potentially limiting prey availability for other piscivorous birds [53]. Greater black-blacked (*Larus marinus*) and herring gulls (*Larus argentatus*) are important nest predators of common tern (*Sterna hirundo*) and least tern (*Sterna antillarum*) nests, and growing populations of these species are thought to have contributed to the decline and abandonment of tern colonies in Chesapeake Bay [52]. In addition, herbivores such as Canada goose and mute swan may overgraze wetland vegetation and deteriorate nesting habitat for marsh-breeding birds. Moreover, guano from these species is rich in nitrogen and phosphorus, and large aggregations have been linked to changes in water chemistry that promote local eutrophication, although only in small water bodies [54].

A critical challenge faced by restoration programs is to decide on criteria for management targets [55]. The ability of our model to describe spatial and short-term temporal variation in estuarine condition suggests a strategy for defining such targets using existing monitoring data. Many of the data analyzed in our research are also collected in a number of estuarine monitoring programs, yet statistical analyses typically are done using traditional ordination or regression approaches that may not be conducive to tracking long-term change in covariance relationships among ecological variables and a large set of potential stressors. Where long time series data are available, SEM or other casual models could be used in conjunction with techniques such as changepoint analysis to identify critical historical thresholds in estuarine condition that could then be used as targets for restoration or mitigation.

Our findings also may contribute to adaptive management of estuarine waterbird communities. As in many coupled natural-human systems, management planning in Chesapeake Bay and other estuaries requires iterated decision making in the face of uncertainty about future changes in land cover and climate [56]. Restoring and maintaining fish and wildlife populations is mandated by state and federal management agencies, but current monitoring in Chesapeake Bay assigns a separate grade to each part of the system and therefore is not entirely consistent with the decision context [57]. Our results provide a snapshot of the chief pathways through which land cover and water quality interact to limit waterbird communities under two extremes of annual rainfall. By themselves, these findings may not cover a sufficient time scale to immediately facilitate management decisions; however, the strong linkages we illustrate among waterbirds, water quality, and land cover indicate the need for a long-term monitoring program to explicitly track the structure of these relationships. Such an approach could help align monitoring efforts with management decision-making and potentially facilitate the knowledge feedback that is central to adaptive management.

## Supporting Information

Figure S1 Results of a generalized additive model showing the relationship between percent urban development in the watershed and the partial residuals of nitrate-N concentration (adjusted for percent cropland) during (A) the drought year of 2002 (*r2* = 0.61, *P* = 0.001) and (B) the wet year of 2003 (*r2* = 0.62, *P* = 0.001). Dashed lines depict 95% confidence intervals.
(TIF)

Text S1 Methods of WCC index development.
(DOCX)

Table S1 List of bird species detected on transect surveys in Chesapeake Bay, USA subestuaries in the dry year of 2002 and the wet year of 2003 and their waterbird community composition (WCC) scores.
(DOCX)

Table S2 Pearson's correlations (below diagonal), variances (diagonal; in bold), and covariances (above diagonal) for variables used in the best-fit structural equation model (SEM) in which all paths were free to vary between the drought year of 2002 and the wet year of 2003.
(DOCX)
