# Supplementary figures and images for: Land Cover and Rainfall Interact to Shape Waterbird Community Composition
Source: PLoS One. 2012 Apr 27;7(4):e35969. doi: 10.1371/journal.pone.0035969 (PMC3338777; doi:10.1371/journal.pone.0035969)

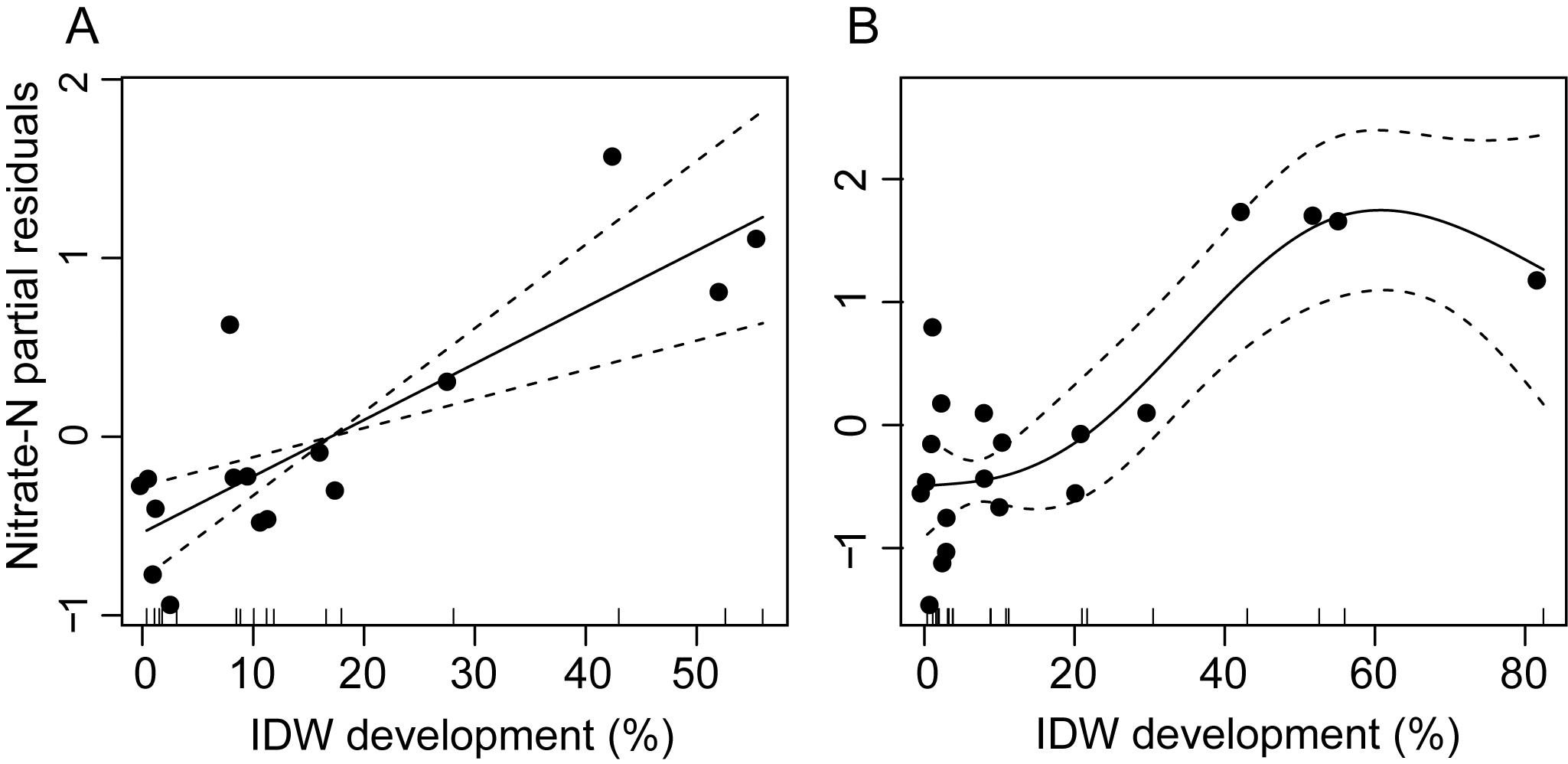

Supplement: Figure S1 — Results of a generalized additive model showing the relationship between percent urban development in the watershed and the partial residuals of nitrate-N concentration (adjusted for percent cropland) during (A) the drought year of 2002 (r2 = 0.61, P = 0.001) and (B) the wet year of 2003 (r2 = 0.62, P = 0.001). Dashed lines depict 95% confidence intervals. (TIF) [file pone.0035969.s001.tif]
