# Supplementary material for: Land Cover and Rainfall Interact to Shape Waterbird Community Composition
Source: PLoS One. 2012 Apr 27;7(4):e35969. doi: 10.1371/journal.pone.0035969 (PMC3338777; doi:10.1371/journal.pone.0035969)
Supplement: Text S1 — Methods of WCC index development. (DOCX) [file pone.0035969.s002.docx]

Text S1

To develop the index of waterbird community integrity (IWCI ) we first calculated a score *S_IWCI_* for each species detected during the study as


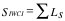


where *L_S_* is the cumulative score of six attributes on a scale of 1 (generalist) to 4 (specialist): (1) foraging niche breadth; (2) nesting sensitivity; (3) migratory status; (4) breeding range; (5) state conservation listing; and (6) native or non-native status. Scores for each species ranged from 5 to 21. Species with scores <10 were considered disturbance tolerant generalists whereas those with scores >10 were considered specialists more sensitive to disturbance.

We used abundance estimates corrected for imperfect detection probabilities to calculate an abundance index *A_I_* for each species along each transect. Corrected abundance estimates across all transects were first divided into quartiles and each was scored based on its placement within those quartiles. Disturbance tolerant species received a higher score of 3 or 4 if their abundance was within the lower quartiles and a low score of 1 or 2 if their abundance was in the upper quartiles. Conversely, disturbance sensitive species were scored higher for abundances in the upper quartiles and lower for abundances in the lower quartiles. *A_I_* was calculated for each transect by taking the mean species abundance score for that transect.

A composite score for each transect *T_IWCI_* was calculated as


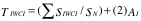


where *S_N_* is the total number of species detected at a transect. *A_I_* was doubled to give it comparable weight to other variables in the equation. A score for the entire subestuary

*E_IWCI_* was calculated by taking the mean of the three *T_IWCI_* scores within a subestuary.
