# Supplementary material for: Land Cover and Rainfall Interact to Shape Waterbird Community Composition
Source: PLoS One. 2012 Apr 27;7(4):e35969. doi: 10.1371/journal.pone.0035969 (PMC3338777; doi:10.1371/journal.pone.0035969)
Supplement: Table S1 — List of bird species detected on transect surveys in Chesapeake Bay, USA subestuaries in the dry year of 2002 and the wet year of 2003 and their waterbird community composition (WCC) scores. (DOCX) [file pone.0035969.s003.docx]

Table S1. List of bird species detected on transect surveys in Chesapeake Bay subestuaries in 2002 and 2003 and their waterbird community composition (WCC) scores.

| **Alpha code** | **Common name** | **Scientific name** | **WCC Score** |
| --- | --- | --- | --- |
| PBGR  DC | Pied-billed grebe | *Podilymbus podiceps* | 10.5 |
| DCCO | Double-crested cormorant | *Phalocrocorax auritus* | 9.5 |
| GBHE | Great blue heron | *Ardea herodias* | 11 |
| GREG | Great egret | *Ardea alba* | 10.5 |
| SNEG | Snowy egret | *Egretta thula* | 11.5 |
| GRHE | Green heron | *Butorides virescens* | 11 |
| MUSW | Mute swan | *Cygnus olor* | 8.5 |
| CAGO | Canada goose | *Branta canadensis* | 7 |
| WODU | Wood duck | *Aix sponsa* | 16.5 |
| MALL | Mallard | *Anas platytyrhyncos* | 7 |
| DODU | Domestic duck | *Anas spp.* | 5 |
| BAEA | Bald eagle | *Haliaetus leucocephalus* | 16.5 |
| OSPR | Osprey | *Pandion haliaetus* | 13 |
| SPSA | Spotted sandpiper | *Actitis malcularia* | 13.5 |
| LAGU | Laughing gull | *Larus atricilla* | 15.5 |
| RBGU | Ring-billed gull | *Larus delewarinsis* | 8.5 |
| HEGU | Herring gull | *Larus argentatus* | 8.5 |
| GBGU | Great black-backed gull | *Larus marinus* | 13.5 |
| ROTE | Royal tern | *Sterna maxima* | 21 |
| COTE | Common tern | *Sterna hirundo* | 16 |
| FOTE | Forster’s tern | *Sterna forsteri* | 14.5 |
| LETE | Least tern | *Sterna antillarum* | 19.5 |
| BEKI | Belted kingfisher | *Ceryle alcyon* | 13 |
