# Supplementary material for: Land Cover and Rainfall Interact to Shape Waterbird Community Composition
Source: PLoS One. 2012 Apr 27;7(4):e35969. doi: 10.1371/journal.pone.0035969 (PMC3338777; doi:10.1371/journal.pone.0035969)
Supplement: Table S2 — Pearson's correlations (below diagonal), variances (diagonal; in bold), and covariances (above diagonal) for variables used in the best-fit structural equation model (SEM) in which all paths were free to vary between the drought year of 2002 and the wet year of 2003. (DOCX) [file pone.0035969.s004.docx]

Table S2. Pearson’s correlations (below diagonal), variances (diagonal; in bold), and covariances (above diagonal) for variables used in the best-fit structural equation model in which all paths were free to vary between the drought year of 2002 and the wet year of 2003.

| **Year** |  | **Surface**  **salinity** | **Percent**  **cropland** | **IDW**  **development** | **Percent**  **marsh** | **DO**  **difference** | **Nitrate-N** | **WCC** |
| --- | --- | --- | --- | --- | --- | --- | --- | --- |
| **2002** | **Surface**  **salinity** | **21.272** | -5.448 | -32.426 | 4.221 | -26.521 | -150.133 | 3.184 |
|  | **Percent**  **cropland** | -0.047 | **606.884** | -234.083 | 18.884 | -183.226 | -163.696 | 18.356 |
|  | **IDW**  **development** | -0.393 | -0.536 | **313.901** | -18.804 | 220.184 | 526.737 | -24.017 |
|  | **Percent**  **marsh** | 0.143 | 0.121 | -0.168 | **39.982** | -27.678 | -13.645 | 5.493 |
|  | **DO**  **difference** | -0.320 | -0.418 | 0.698 | -0.246 | **317.198** | 285.126 | -19.937 |
|  | **Nitrate-N** | -0.617 | -0.127 | 0.570 | -0.041 | 0.307 | **2722.589** | -24.902 |
|  | **WCC** | 0.360 | 0.393 | -0.715 | 0.458 | -0.590 | -0.252 | **3.595** |
|  |  | **Surface**  **salinity** | **Percent**  **cropland** | **IDW**  **development** | **Percent**  **marsh** | **DO**  **difference** | **Nitrate-N** | **WCC** |
| **2003** | **Surface**  **salinty** | **6.562** | -15.386 | -18.004 | 12.100 | -8.887 | -433.045 | 1.354 |
|  | **Percent**  **cropland** | -0.258 | **542.217** | -245.029 | 25.842 | -217.987 | 1046.746 | 8.589 |
|  | **IDW**  **development** | -0.313 | -0.469 | **504.284** | -59.063 | -59.063 | 2299.101 | -16.818 |
|  | **Percent**  **marsh** | 0.414 | 0.097 | -0.231 | **129.961** | -63.903 | -284.635 | 4.815 |
|  | **DO**  **difference** | -0.197 | -0.533 | 0.519 | -0.319 | **308.331** | -983.643 | -9.163 |
|  | **Nitrate-N** | -0.709 | 0.188 | 0.429 | -0.105 | 0.236 | **56900.431** | -124.937 |
|  | **WCC** | 0.563 | 0.393 | -0.768 | 0.450 | -0.556 | -0.558 | **0.880** |
